# Mucogingival Parameters of Maxillary Anterior Teeth in Peruvian Dental Students: A Cross-Sectional Study

**DOI:** 10.21142/2523-2754-1304-2025-261

**Published:** 2025-11-08

**Authors:** Elizabeth Kalett Jiménez Fabián, Lisseth Modesta Alarcón Rosas, Consuelo Marroquín-Soto, César-Augusto Padilla-Avalos

**Affiliations:** 1 School of Dentistry, Universidad Cientifica del Sur. Lima, Peru. 100073080@cientifica.edu.pe 100030745@cientifica.edu.pe Lima Peru 100073080@cientifica.edu.pe 100030745@cientifica.edu.pe; 2 Associate Researcher, School of Dentistry, Universidad Cientifica del Sur. Lima, Peru. cmarroquins@cientifica.edu.pe Lima Peru cmarroquins@cientifica.edu.pe; 3 Research Professor, Research and Innovation Laboratory in Digital Dentistry, School of Dentistry, Universidad Cientifica del Sur. Lima, Peru. cpadillaav@cientifica.edu.pe Lima Peru cpadillaav@cientifica.edu.pe

**Keywords:** Periodontics, Periodontium, Gingiva, Oral Health, periodoncia, periodonto, encía, salud oral

## Abstract

**Objective:**

To determine the mucogingival parameters of the maxillary anterior teeth in Peruvian dental students, including the width of keratinized gingiva, gingival phenotype, interdental papilla height, and gingival zenith position**.**

**Materials and Methods:**

A cross-sectional study was conducted among 128 randomly selected dental students from a private university in Lima, Peru. Prior to data collection, a pilot study and examiner calibration were performed. Mucogingival parameters were clinically assessed using a North Carolina periodontal probe. Measurements were analyzed by tooth type, sex, and age using the Chi-square test, Mann-Whitney U test, Friedman test, and Dunn’s post hoc test (α = 0.05).

**Results:**

The mean width of keratinized gingiva ranged from 4.99 mm to 5.29 mm, and the height of the interdental papilla ranged from 3.99 mm to 4.41 mm. A thick gingival phenotype was observed in 83.6% of participants. Gingival zenith deviation from the midline ranged from 0.15 mm to 0.18 mm. No statistically significant differences in mucogingival parameters were found by sex or age.

**Conclusion:**

Mucogingival parameters showed notable individual variability among young Peruvian dental students, with no significant influence of sex or age. These values may serve as clinical reference points for periodontal assessment in the esthetic zone. Clinical relevance highlighted that population-specific data on mucogingival characteristics can improve diagnostic accuracy and support personalized treatment planning. These findings contribute to clinical decision-making and future research in periodontology and esthetic dentistry.

## RESUMEN

**Objetivo:**

Determinar los parámetros mucogingivales de los dientes anteriores maxilares en estudiantes de odontología peruanos, incluyendo el ancho de encía queratinizada, el fenotipo gingival, la altura de la papila interdental y la posición del cénit gingival.

**Materiales y métodos:**

Se realizó un estudio transversal en 128 estudiantes de odontología seleccionados aleatoriamente de una universidad privada en Lima, Perú. Antes de la recolección de datos, se llevó a cabo un estudio piloto y la calibración de los examinadores. Los parámetros mucogingivales fueron evaluados clínicamente utilizando una sonda periodontal tipo Carolina del Norte. Las mediciones se analizaron por tipo de diente, sexo y edad mediante las pruebas de Chi-cuadrado, Mann-Whitney U, Friedman y post hoc de Dunn (α = 0,05).

**Resultados:**

El ancho promedio de encía queratinizada osciló entre 4,99 y 5,29 mm, y la altura de la papila interdental entre 3,99 y 4,41 mm. Se observó un fenotipo gingival grueso en el 83,6% de los participantes. La desviación del cénit gingival respecto a la línea media varió de 0,15 mm a 0,18 mm. No se encontraron diferencias estadísticamente significativas en los parámetros mucogingivales según el sexo o la edad.

**Conclusiones:**

Los parámetros mucogingivales mostraron una notable variabilidad individual entre los estudiantes de odontología peruanos jóvenes, sin influencia significativa del sexo o la edad. Estos valores pueden servir como referencias clínicas para la evaluación periodontal en la zona estética. La relevancia clínica señala que los datos específicos de la población sobre características mucogingivales pueden mejorar la precisión diagnóstica y respaldar una planificación de tratamiento personalizada. Estos hallazgos contribuyen a la práctica clínica y a futuras investigaciones en periodoncia y odontología estética.

## INTRODUCTION

Mucogingival parameters are key clinical indicators that describe the relationship between the gingiva and alveolar mucosa. Assessing both the quantity and quality of gingival tissue plays a vital role in periodontal diagnosis and treatment planning, particularly in procedures aimed at achieving esthetic harmony and functional stability ^1^^,^^2^. In the anterior region, where patient expectations are increasingly high, the success of esthetic rehabilitations depends not only on tooth position but also on the symmetry, contour, and integration of the surrounding gingival tissues, commonly referred to as “pink esthetics” ^3^^,^^4^.

Several investigations have examined the morphological characteristics of gingival tissues in the anterior zone, highlighting the influence of variables such as sex and age ^5^. In contrast, other studies have focused on younger populations, using diverse clinical assessment techniques, and have emphasized that the available evidence remains limited, particularly for certain ethnic or geographic groups ^6^^,^^7^.

Among the most commonly evaluated parameters, the width of keratinized gingiva is particularly important for establishing prognosis and selecting surgical or restorative approaches. This width is measured from the gingival margin to the mucogingival junction using a periodontal probe ^8^. Another essential parameter is the gingival phenotype, which has gained prominence due to its influence on esthetic outcomes. Typically, it is assessed by evaluating the transparency of a periodontal probe through the gingival margin and classifying the tissue as either thin or thick ^2^^,^^9^. Furthermore, studies have proposed a potential correlation between gingival phenotype and keratinized gingiva width ^10^^,^^11^.

The height and presence of the interdental papilla also significantly contribute to both function and smile esthetics. The absence of this papilla may result in “black triangles,” which compromise phonetics, increase plaque retention, and predispose to caries and food impaction ^12^. Similarly, the gingival zenith, the most apical point of the gingival margin, is a critical landmark in achieving optimal periodontal and prosthetic results in the anterior region ^13^.

In addition to objective parameters, the patient’s perception of gingival esthetics has become a relevant factor in treatment acceptance and satisfaction ^14^. Therefore, a detailed understanding of the dentogingival complex, particularly in the maxillary anterior region, is essential to achieve a harmonious smile design ^15^^,^^16^.

The clinical relevance of studying mucogingival parameters lies in their value as baseline data for interdisciplinary esthetic planning. However, despite their significance, few studies have characterized these parameters in Latin American populations. Given the limited literature available, it is necessary to describe the mucogingival characteristics of specific populations, such as young university students, to provide reference values that support both diagnosis and treatment. Therefore, the aim of this study was to describe the mucogingival parameters of the upper anterior teeth in a cohort of Peruvian dental undergraduates.

## METHODS

### Ethical Considerations

The study was approved by the Institutional Ethics Committee (CIEI) of Universidad Científica del Sur® (Approval No. 296-CIEI-CIENTÍFICA-2022). All participants provided written informed consent prior to participation, in accordance with the Declaration of Helsinki and institutional regulations to ensure autonomy and data confidentiality.

### Study Design and Setting

This was an observational, analytical, cross-sectional, and prospective study conducted among undergraduate dental students at a private university in Lima, Peru. The total population consisted of 191 students enrolled in clinical academic years.

### Sample Design

The sample size was calculated using Epidat® version 4.2, assuming a 50% expected proportion (for unknown population parameters), a 95% confidence level, and a 5% margin of error. This calculation yielded a minimum required sample of 128 students, who were randomly selected from the target population.

### Participant Selection Criteria

Participants included undergraduate dental students aged 19 to 28 years. Exclusion criteria included students undergoing active orthodontic or prosthodontic treatment, as well as those with structural anomalies in the upper anterior teeth (such as alterations in shape, size, or position due to trauma or pathology).

### Data Collection

#### Pilot Study and Examiner Calibration

Before clinical data collection, two examiners were calibrated under the supervision of an experienced periodontist. Calibration was performed on a pilot sample of 10 students. Cohen's Kappa coefficients were 0.80 (inter-examiner) and 1.00 (intra-examiner), and the intraclass correlation coefficient (ICC) was 1.00, confirming excellent measurement reliability.

#### Clinical Examination

**Figure 1:**
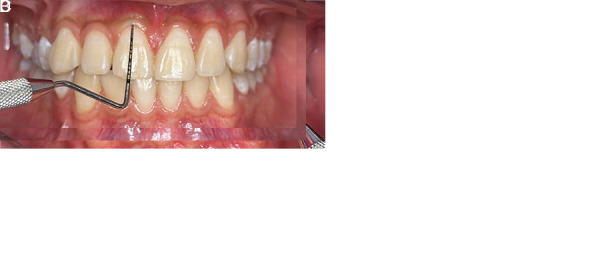
Clinical assessment of mucogingival parameters in the maxillary anterior region. A. Measurement of the width of keratinized gingiva using a North Carolina periodontal probe, from the free gingival margin to the mucogingival junction. B. Assessment of interdental papilla height, measured from the papilla tip to an imaginary line connecting the mid-facial cementoenamel junctions of adjacent teeth. C. Evaluation of gingival phenotype by probe transparency through the gingival sulcus: visibility indicates a thin phenotype; non-visibility indicates a thick phenotype. D. Determination of the gingival zenith, recorded as the most apical point of the gingival margin relative to the longitudinal axis of each anterior tooth.

Periodontal evaluation focused on the buccal surfaces of the maxillary anterior teeth (central incisors, lateral incisors, and canines). The following mucogingival parameters were assessed using a North Carolina periodontal probe (Hu-Friedy®, Chicago, USA):

• Width of keratinized gingiva: measured as the distance (mm) from the gingival margin to the mucogingival junction at the mid-buccal site (Fig. 1A).
• Interdental papilla height: measured as the vertical distance from the papilla tip to an imaginary line connecting the mid-facial cemento-enamel junctions (CEJs) of adjacent teeth (Fig. 1B).
• Gingival phenotype: determined by probe translucency at the sulcus; classified as thin (probe visible) or thick (probe not visible) (Fig. 1C).
• Gingival zenith: recorded as the most apical point of the gingival margin in relation to the vertical midline of each tooth (Fig. 1D).

### Statistical Analysis

Data were processed using SPSS® version 25.0 (IBM, Armonk, NY, USA), with the significance level set at p < 0.05. The Kolmogorov-Smirnov test was used to evaluate data normality. The Chi-square test assessed associations between categorical variables. The Mann-Whitney U test was used to compare sex and age groups, while the Kruskal-Wallis test evaluated differences between tooth types. When applicable , Dunn’s post hoc test was used to identify specific intergroup differences.

## RESULTS

The total sample included 128 undergraduate dental students, of whom 81 (63%) were female and 47 (37%) were male. Participants were grouped into two age groups: 19-23 years (n = 86) and 24-28 years (n = 42).

### Keratinized Gingiva Width

Table 1 presents the mean widths of keratinized gingiva across the maxillary anterior teeth. Statistically significant differences were observed between sexes in teeth 1.1 (p = 0.016) and 1.2 (p = 0.018), with males showing greater widths. However, no significant differences were noted by age groups for any tooth (p > 0.05). When assesing overall keratinized tissue width across all anterior teeth, no statistically significant association was found with sex (p = 0.120) or age (p = 0.300).

**Table:** 

Tooth	Sex					Age (yrs)					Total	
	Females		Males		P-value	19 to 23		24 to 28		P				
	Mean	SD	Mean	SD		Mean	SD	Mean	SD		Mean	SD
1.1	4.88^a^	1.07	5.28^a^	0.95	0.016	4.90^a^	0.96	5.29^a^	1.15	0.064	5.02^a^	1.04
1.2	5.04^a^	0.94	5.45^a^	1.10	0.018	5.14^a^	1.04	5.29^a^	0.97	0.381	5.19^a^	1.02
1.3	5.28^a^	0.96	5.30^a^	0.95	0.970	5.24^a^	0.96	5.38^a^	0.96	0.520	5.29^a^	0.96
2.1	5.02^a^	1.01	5.21^a^	0.81	0.138	5.07^a^	0.92	5.14^a^	1.00	0.919	5.09^a^	0.94
2.2	5.04^a^	0.91	5.32^a^	1.11	0.183	5.07^a^	0.99	5.29^a^	0.99	0.231	5.14^a^	0.99
2.3	4.93^a^	1.16	5.11^a^	0.98	0.211	4.93^a^	1.13	5.12^a^	1.04	0.390	4.99^a^	1.10
p-value	0.120		0.734			0.300		0,830			0,291		

SD, Standard deviation. * Mann-Whitney U test. Kruskal-Wallis H test. Different letters indicate statistically significant differences using Post-Hoc with Bonferroni correction.

### Interdental Papilla Height

Table 2 shows interdental papilla heights. A significant difference by tooth site was found in female participants (p = 0.005). Additionally, a significant difference by age was detected (p = 0.031), with higher papilla heights noted in the 19-23 age group, particularly between teeth 1.2-1.1 (p = 0.038). However, no significant differences were observed overall by sex (p = 0.188).

**Table 2:** Parameters of interdentary papilla in millimeters of the upper anterior teeth according to sex and age.

Tooth	Sex					Age (yrs)					Total	
	Females		Males		P-value	19 to 23		24 to 28		P				
	Mean	SD	Mean	SD		Mean	SD	Mean	SD		Mean	SD
1.3 - 1.2	3.94^a^	0.89	4.09^a^	0.86	0.380	4.02^a^	0.85	3.93^a^	0.92	0.428	3.99^a^	0.87
1.2 - 1.1	4.17^abc^	0.63	4.26^a^	0.71	0.460	4.29^c^	0.65	4.02^a^	0.64	0.038	4.20^b^	0.66
1.1 - 2.1	4.36^b^	0.81	4.51^a^	0.75	0.263	4.41^bc^	0.82	4.43 ^b^	0.74	0.713	4.41^c^	0.79
2.1 - 2.2	4.36^bc^	0.75	4.30^a^	0.86	0.587	4.40^bc^	0.82	4.21^ab^	0.72	0.332	4.34^b^	0.79
2.2 - 2.3	4.20^bc^	0.77	4.28^a^	1.06	0.621	4.23^ab^	0.85	4.21^ab^	0.95	0.893	4.23^b^	0.88
p-value	0.005		0.188			0.031		0.023			‹0.001		

SD, standard deviation. *Mann-Whitney U test.

Kruskal-Wallis H test. Different letters indicate statistically significant differences using Post-Hoc with Bonferroni correction.

### Gingival Phenotype

As summarized in Table 3, the thick gingival phenotype was dominant in the study population, observed in 83.6% of participants. There were no statistically significant differences in phenotype distribution by sex (p = 0.886) or age group (p = 0.573). 

**Table 3:** Parameters of gingival phenotype in millimeters of the upper anterior teeth according to sex and age.

Gingival phenotype	Sex				Age (yrs)				Total	
	Females		Males		19 to 23		24 to 28					
	f	%	f	%	f	%	f	%	f	%
Thin	13	10.2%	8	6.3%	13	10.2%	8	6.3%	21	16.4%
Thick	68	53.1%	39	30.5%	73	57.0%	34	26.6%	107	83.6%
*p*-value		0.886				0.573				

* Pearson Chi-square test

### Gingival Zenith

Table 4 details the gingival zenith measurements. No significant differences were found by sex for any tooth (p ≥ 0.951). However, for tooth 1.3, the 24-28 age group showed significantly greater zenith values (p = 0.030). Overall, no significant associations were found between zenith measurements and sex (p = 0.951), age (p = 0.929), or total sample (p = 0.975).

**Table 4:** Parameters of gingival zenith in millimeters of the upper anterior teeth according to sex and age.

Tooth	Sex					Age (years)					Total	
	Female		Male		P	19 to 23		24 to 28		P				
	Mean	SD	Mean	SD		Mean	SD	Mean	SD		Mean	DS
1.1	0.17^a^	0.38	0.17^a^	0.38	0.970	0.17^a^	0.38	0.17^a^	0.38	0.913	0.17^a^	0.38
1.2	0.16^a^	0.37	0.15^a^	0.36	0.863	0.17^a^	0.38	0.12^a^	0.33	0.420	0.16^a^	0.36
1.3	0.20^a^	0.40	0.15^a^	0.36	0.492	0.13^a^	0.34	0.29^a^	0.46	0.030	0.18^a^	0.39
2.1	0.17^a^	0.38	0.11^a^	0.31	0.310	0.14^a^	0.35	0.17^a^	0.38	0.686	0.15^a^	0.36
2.2	0.21^a^	0.41	0.13^a^	0.34	0.245	0.15^a^	0.36	0.24^a^	0.43	0.231	0.18^a^	0.39
2.3	0.16^a^	0.37	0.15^a^	0.36	0.863	0.17^a^	0.38	0.12^a^	0.33	0.420	0.16^a^	0.36
*P* value	0.951		0.968			0.929			0.283			0.975	

SD, standard deviation. *Mann-Whitney U test. Kruskal-Wallis H test. Different letters indicate statistically significant differences using Post-Hoc with Bonferroni correction.

## DISCUSSION

The evaluation of mucogingival parameters in the upper anterior region is fundamental for accurate diagnosis and for planning periodontal and esthetic treatments. Biological variability related to sex and age should be considered, especially in clinical procedures where symmetry and soft tissue harmony are critical. Therefore, the present study aimed to assess the width of keratinized gingiva, interdental papilla height, gingival phenotype, and gingival zenith position according to sex and age in a population of young Peruvian dental students aged 19 to 28 years.

Achieving an esthetically pleasing and functionally stable smile requires precise balance in the position and symmetry of the supporting soft tissues ^17^. Among these, keratinized gingiva is a critical factor for the long-term maintenance of periodontal health and is often used as a reference for therapeutic decision-making in mucogingival procedures ^8^. The width of keratinized tissue and the periodontal phenotype are especially relevant in both surgical and restorative disciplines, as they directly affect treatment predictability ^10^.

The 2017 World Workshop on the Classification of Periodontal and Peri-Implant Diseases highlighted that the width of keratinized tissue varies according to gingival biotype, reporting ranges from 2.75 to 5.44 mm for thin phenotypes and from 5.09 to 6.65 mm for thick ones ^1^. In the current study, the width of keratinized gingiva in the upper anterior teeth ranged from 4.99 ± 1.10 mm to 5.29 ± 0.96 mm, which falls within these reference values. A significant difference was observed between sexes in teeth 1.1 and 1.2 (p = 0.016 and p = 0.018, respectively), with greater values in males. These findings support previous consensus reports that identified a positive relationship between gingival thickness and keratinized tissue width in the anterior maxilla ^18^.

Interdental papilla height is another critical factor in periodontal and esthetic evaluation. This soft tissue structure contributes not only to the harmony of the smile but also to the preservation of interdental health ^19^. Loss of papillary height, often seen in elderly or periodontally compromised individuals, has been associated with functional and esthetic concerns ^12^. Although several variables may influence papillary dimensions, some studies have shown that a thin gingival phenotype alone may not be a determining factor for papilla loss ^20^. In the current study, the mean height of the interdental papilla ranged from 3.99 ± 0.87 mm to 4.41 ± 0.79 mm, slightly higher than values previously reported in other populations (3.85 mm mesial, 3.9 mm distal) ^21^. A statistically significant association was observed between tooth site and papillary height in females (p = 0.005), as well as between age group and papillary height (p = 0.031), with greater values in the 24-28 age group (p = 0.023).

Various techniques have been proposed to assess gingival thickness, including the use of rubber-tipped endodontic instruments under local anesthesia ^9^. However, probing transparency through the sulcus remains a simple and reliable clinical method to distinguish between thin and thick phenotypes ^20^. In the present study, this method was used to classify gingival phenotype, revealing a predominance of the thick phenotype in 83.6% of the participants. This result aligns with previous studies indicating that maxillary central incisors exhibit the greatest gingival thickness, followed by lateral incisors and canines ^18^, and with reports that found a similar prevalence of thick phenotype (76%) in individuals aged 20 to 35 years ^22^. No statistically significant associations were observed between gingival phenotype and sex or age (p = 0.886 and p = 0.573, respectively).

The gingival zenith serves as a fundamental esthetic reference for smile design. Both digital and conventional methods have been used to assess its position ^13^. In a classic study by Chu et al., the gingival zenith of central incisors was located approximately 1 mm distal to the vertical midline, about 0.4 mm in lateral incisors, and aligned with the midline in canines ^23^. In our analysis, the mean zenith deviation ranged from 0.15 to 0.21 mm, with no significant differences by sex (p ≥ 0.951). A statistically significant association was found for tooth 1.3 (p = 0.030), with slightly higher zenith values in the 24-28 age group. However, overall, no significant variation in zenith position was observed in the total sample (p = 0.975).

In summary, this study found that the width of keratinized gingiva and interdental papilla height exhibited significant differences according to sex and age, while gingival phenotype and gingival zenith did not. These findings align with previous literature but also underscore the importance of considering population-specific characteristics when establishing diagnostic and treatment parameters.

This analysis provides valuable data on mucogingival parameters in young Peruvian individuals, providing reference values for clinical decision-making in periodontal and esthetic contexts. While the results are consistent with international findings, they emphasize the importance of population-specific assessment. Further studies with larger and more diverse samples are recommended to validate these results and support the development of personalized treatment protocols.

This cross-sectional study was conducted in a defined population with standardized clinical parameters assessed under calibrated conditions. The scope of the analysis was limited to clinical evaluation of mucogingival characteristics in the maxillary anterior region, without inclusion of radiographic or digital measurements. The variables analyzed were confined to age and sex, as the primary focus was to establish baseline clinical references. Future research may explore broader variables or incorporate advanced tools for complementary assessment.

## CONCLUSIONS

The assessment of mucogingival parameters in the maxillary anterior region is fundamental for esthetic evaluation and treatment planning. These parameters provide valuable clinical reference values and facilitate the identification of common patterns within specific populations. In this study, although certain statistically significant differences were observed in the width of keratinized gingiva and interdental papilla height according to sex and age, no such associations were found for gingival phenotype or gingival zenith position. Overall, mucogingival characteristics demonstrated considerable individual variability.

While these results reflect the profile of a well-defined population of young Peruvian dental undergraduates, they may be influenced by ethnic background, facial biotype, or genetic traits. Therefore, comparisons with other populations should take these contextual variables into account. The findings underscore the importance of incorporating population-specific data into periodontal and esthetic treatment protocols to enhance clinical precision and predictability.

Further research involving larger, more diverse, and multicenter populations is recommended to deepen our understanding of mucogingival variations across different age groups, sexes, and ethnic backgrounds. Such studies will support the development of evidence-based guidelines for personalized periodontal and restorative therapies.
